# Updates on mechanistic insights and targeting of tumour metastasis

**DOI:** 10.1111/jcmm.14931

**Published:** 2020-01-19

**Authors:** Zeru Feng, Qiuxuan Yu, Ting Zhang, Wanpeng Tie, Jing Li, Xikun Zhou

**Affiliations:** ^1^ State Key Laboratory of Oral Diseases National Clinical Research Center for Oral Diseases Chinese Academy of Medical Sciences Research Unit of Oral Carcinogenesis and Management West China College of Stomatology Sichuan University Chengdu China; ^2^ State Key Laboratory of Biotherapy and Cancer Center West China Hospital Collaborative Innovation Center for Biotherapy Sichuan University Chengdu China

**Keywords:** metastasis, tumour invasion, tumour microenvironment

## Abstract

Malignant tumours are one of the major diseases that seriously endanger human health. The characteristics of their invasion and metastasis are one of the main causes of death in cancer patients, and these features cannot be separated from the participation of various molecules‐related cells living in the tumour microenvironment and specific structures. Tumour invasion can approximately be divided into several specific steps according to the movement of tumour cells. In each step, there are different actions in the tumour microenvironment that mediate the interactions among substances. Researchers are attempting to clarify every mechanism of the tumour dissemination. However, there is still a long way to the final determination. Here, we review these interactions in tumour invasion and metastasis at the structural, molecular and cellular levels. We also discuss the ongoing studies and the promise of targeting metastasis in tumour therapy.

## 1 INTRODUCTION

At present, research on tumours is in full swing. Primary tumours can be treated by surgical resection, but cancer mortality increases significantly once the tumour metastasizes. Tumour metastasis, as an important signal of cancer staging, has become a hot topic in cancer treatment. Therefore, research on tumour invasion and metastasis is particularly important. The metastasis caused by carcinomas is formed following the completion of a complex succession of cell‐biological events, collectively termed the invasion‐metastasis cascade.^1^ In this process, there are not only related oncogenes, tumour suppressor genes, tumour metastasis‐associated genes and related factors (adhesion‐related molecules, angiogenesis factors, signal transduction molecules, proteolytic enzymes, matrix metalloproteinases, etc) but also various biological structures, such as tumour blood vessels and adhesion structures. The activities of various tumour metastasis‐related molecules and the formation of various biological structures bounding closely with each other throughout the whole process finally complete the tumour dissemination.

The tumour microenvironment, where tumour cells live, includes a variety of cells (such as cancer‐associated fibroblasts (CAFs), tumour‐associated macrophages (TAMs), cancer stem cells (CSCs) and endothelial cells) and extracellular matrix proteins that are predominant in tumour metastasis invasion.^2^ There are distinct variable relationships among all the components. Most substances can promote tumour metastasis and, in return, some aspects of these components change beyond the influence of the tumour microenvironment.^3^ To some extent, the changes at each level are definitely not parallel, with several cross points that provide an immense network for new target therapy in tumour care. This review describes recent findings on the mechanisms of how these associated components convert their roles and the different activities occurring afterwards according to the chronological sequence of invasion.

## 2 STAGE OF TUMOUR PROGRESSION

At present, the TNM staging system is the most widely used staging system in the world.^4^ The TNM staging system is based on the local growth (T), lymph node metastasis (N) and distant metastasis (M) of the tumour. A tumour has four T stages, three N stages and two M stages, with a total of 24 TNM combinations. There are multiple classification methods for each site: clinical classification is represented by cTNM or TNM, pathological classification (pTNM), recurrence classification (rTNM) and autopsy classification (aTNM). cTNM system is essential for the selection and evaluation of initial treatment options. This system is determined before treatment without any subsequent information changes. When patients are no longer treated, clinical staging must be stopped. Pathological staging provides more accurate information on the basis of pretreatment data, and other evidence obtained from surgery (especially pathological diagnosis). In fact, the clinical and pathological classification are combined to make the final judgment. Histological grade divides tumour differentiation into four levels, expressed by the degree of similarity between tumour and normal tissue at the site of invasion. G1 to G4, respectively, represent highly differentiated, medium‐differentiated, low‐differentiated and undifferentiated tumours. There are also specialized abbreviation for other identifiers including lymphatic invasion (L), venous invasion (V) and residual tumour (R).^5^

## 3 STRUCTURAL BASIS OF TUMOUR METASTASIS

As mentioned previously, there are several steps in the invasion‐metastasis cascade: local invasion through the surrounding extracellular matrix (ECM) and stromal cell layers, intravasation into the lamina of blood vessels, surviving the rigours of transport through the vasculature, arresting at distant organ sites, extravasation, surviving the foreign microenvironments to form micro‐metastasis and finally, reviving the proliferative programmes at metastatic sites, thereby generating macroscopic and clinically detectable neoplastic growths.^1^

### 3.1 Local invasion

**Figure 1:**
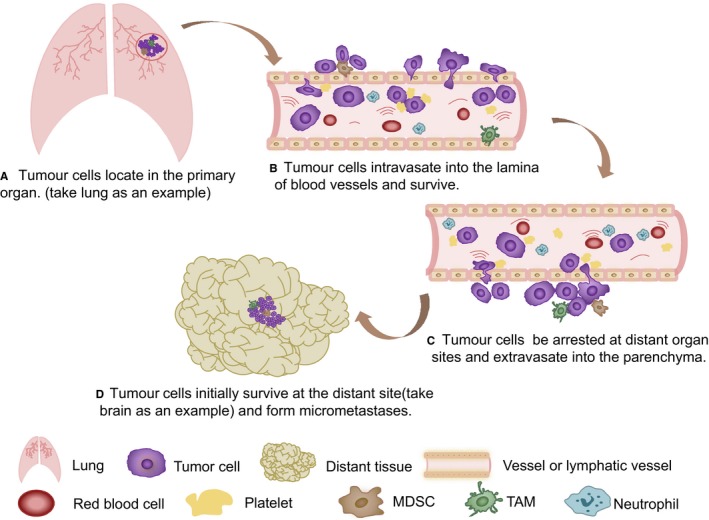
Tumour metastasis cascade. Tumour cells transfer from the primary organ through the vasculature to the target tissue. This process has been divided into four stages in human, that these stages are actually continuous in vivo, without any hesitation or pause. A, Tumour cells invade locally through the surrounding extracellular matrix (ECM) and stromal cell layers where growth factors participate in the degradation of the basement membrane and other ECM components. B, Tumour cells intravasate into the lamina of blood vessels. During their residence in vessels, these cells fight against the primary cells in the vessels and hide from immune substances to survive the rigours of transport through the vasculature. C, Tumour cells can be arrested at distant organ sites by special surface makers and membrane ligands subsequently. This specialty may not be one hundred per cent. D, Tumour cells begin to extravasate into the parenchyma of distant tissues and initially survive in these foreign microenvironments to form micro‐metastasis that collect useful molecules and finally reinitiate their proliferative programmes at metastatic sites, thereby completing the tumour metastasis

The so‐called local invasion is that the cancer cells located in the primary tumour enter the surrounding matrix and then migrate into the adjacent normal tissue parenchyma,^1^ which is closely related to the structure of the basement membrane in the extracellular matrix around the cancer cells (Figure 1A). In addition to the structural function of the basement membrane (separating epithelial cells and stromal cells), the extracellular matrix contains a repository of tumour‐derived lineage growth factors. The basal membrane also functions during the signal transduction of cancer cells through integrin‐mediated initiation of cell‐matrix adhesion, causing changes in cell polarity, proliferation, invasion and survival ability.^6^

Matrix metalloproteinase (MMPs) and epithelial‐mesenchymal transition (EMT) mediate this process. Cells expressing MMPs release growth factors, which are involved in the degradation of the basement membrane as well as other extracellular matrix (ECM) components and promote the proliferation of cancer cells.^7^ The activities of MMPs are carefully regulated by transcriptional and posttranslational mechanisms in normal tissues while cancer cells can decipher the strict control of MMPs activity, maximizing its function. In addition, the connection between epithelial cells is a great obstacle to the local invasion programme. To overcome this barrier, cancer cells dissolute adhesions, eliminate cell polarity, breakdown the cells into individual forms, assisting in epithelial‐mesenchymal metastasis.^8^ Moreover, there is a two‐way interaction between tumour cells and the nearby matrix. Cancer cells stimulate the formation of the inflammatory matrix, thus establishing a positive feedback of self‐amplification.^1^

### 3.2 Intravasation and survival in circulation

Lymphatic and blood diffusion are two ways of infiltration, and the latter seems to be the main mechanism of metastasis.^9^ All solid tumours require a vascular supply in order to progress.^10^ Tumour blood vessels have three characteristics as the structural basis of intravasation as described below (Figure 1B). Firstly, these vessels cause tumour cells to grow rapidly. Unlike blood vessels in normal tissues, the neovascularization produced by cancer cells is tortuous and spirally extended. The spiral vessels are much longer in length, the blood flow volume is larger and the blood pressure is much higher, which triples the normal levels. The above features result in the rush of nutrient absorption. Secondly, too many tumour blood vessels could destroy tissue. The normal growth period of vessels is one year, while the growth cycle of tumour blood vessels is only a few days. Because of the rapid growth, the number of tumour blood vessels can reach hundreds quickly, pulling down the original tissue. Thirdly, there are holes in tumour blood vessels causing pleural effusion and ascites later on. Normal human blood vessels have three layers, as blood vessels of the tumour only have the intima with tiny holes.^11^ The weak interaction between adjacent endothelial cells and the lack of pericyte coverage may promote intravascular dilation and enhance the capability of crossing the barrier between pericytes and endothelial cells, forming microvascular walls.^12^ In addition, tumour endothelial cells (TECs) differ from normal endothelial cells. TECs, grow faster with lower serum requirements, respond to growth factors better, express special genes, have cytogenetic abnormalities and are more resistant to chemotherapeutic drugs. Lately, a new process related to tumour metastasis termed vessel co‐option has been found in human tumours growing in the brain, liver, lungs and lymph nodes. Vessel co‐option is a non‐angiogenic process through which tumour cells utilize pre‐existing tissue blood vessels to support itself, but its adhesion pathways are unknown.^10^

After cancer cells invade the surroundings and intravasate, these cells, named CTCs (circulating tumour cells), are located between the primary tumour and the site of transmission and shoulder the task of surviving under various pressures to reach distant organs. In the absence of integrin‐dependent adhesion to the extracellular matrix component, epithelial cells usually initiate anoikis. Anoikis, a form of apoptosis, is a special programmed cell death induced by the loss of contact between cells and the extracellular matrix.^13^ In addition to the stress caused by matrix stripping, CTCs must overcome hemodynamic shear stress and avoid capture by innate immune system cells. Conveniently, CTCs seem to nimbly elude threats through the relatively large embolus formed by the interaction between tumour cells and platelets. As a result, platelet‐coated tumour cells are able to survive in circulation until they are captured at distant tissue sites.^14^

### 3.3 Being arrested at a distant organ site and extravasation

Although CTCs can theoretically spread to a variety of secondary sites, clinicians have noticed that a single cancer type forms metastatic organ in a limited number of target organs (Figure 1C). There are two assumptions regarding this formative mechanism: one belief claims that it is a passive physical process due to structure. According to this view, the tissue orientation of cancer cells is only a passive process. However, some CTCs may not have this rapid capture due to their unusual plasticity or the chance of shunting through arteriovenous channels, thus enabling these cells to remain in further organs.^15^ The other expects predefined preferences for hosting in some organizations. In fact, some cancer cells can form specific adhesions in particular tissues, which is more conducive to the entrapment of these cancer cells. It has been suggested, for example, that the expression of metadherin in breast cancer cells leads to lung metastasis by promoting the binding of metadherin to its pulmonary vessels.^16^ Alternatively, CTCs can metastasize to specific organs through the interaction of these cells with ligand receptors of the microvascular lumen, whose mechanisms require further study.

After the tumour cells are captured, the next step is to infiltrate into the substance of the distant tissue. Although most models of metastasis include an extravasation step early in the process, study shows that metastasis is mainly induced by the proliferation of tumour cells attached to the vascular endothelium while the extravasation of tumour cells is rare.^17^

In addition, as discussed earlier, the neovascularization of primary tumours is zigzag and leaky. However, microvessels in normal tissues at a distance are normal‐like, resulting in reduced permeability. For example, disseminated cancer cells that attempt to reach the brain's parenchyma must cross the blood‐brain barrier.^18^ Similarly, endothelial cells lining the pulmonary microvessels typically form a large degree of the air‐blood barrier. To overcome the physical barrier of low permeability microvessels in distant normal tissues to the exosmosis of cancer cells, primary tumours can secrete protein angiopoietin and MMPs to interfere with these distant tissues. These factors destroy vascular endothelial cell‐cell connections and promote the overflow of breast cancer cells in the lungs.^19^ In contrast, cancer cells that reach the bone or liver encounter highly permeable sinuses, even under normal conditions, which means little to no tumour cell spills.^20^ In other words, the characteristics of special microenvironments may have a profound impact on the fate of disseminated cancer cells.

### 3.4 Micro‐metastasis formation and metastatic colonization

The microenvironment of the metastatic site usually differs from that of the primary site, which means that cancer cells, at least in the beginning, are not fully adapted to their newly discovered home. The diversity may include the types of stromal cells, the components of the extracellular matrix, the effective cytokines or even the microstructure of the tissue itself (Figure 1D). Some researchers speculate that cancer cells can solve the problem of an incompatible microenvironment by establishing a pre‐metastatic niche.^21^ According to this model, primary tumours release systemic signals, induce fibroblasts in tissues to specifically up‐regulate the secretion of fibronectin and mobilize VEGFR1 positive hematopoietic progenitor cells from bone marrow through homing interactions to these future metastatic sites. These hematopoietic progenitor cells then secrete MMP‐9 to alter the local microenvironment of these loci.^22^, ^23^ Importantly, all these events are thought to occur before the cancer cells reach the metastatic site. In addition, cancer cells can also initiate cellular autonomy, regulating signal transduction pathways.^21^ In the end, the remote microenvironment better suits tumour cells.

If only disseminated cancer cells are first exposed to the microenvironment of foreign tissues and successfully survive, they still do not guarantee proliferation. Conversely, most disseminated tumour cells appear to have experienced slow depletion for several weeks or even months, otherwise to remain dormant as microcolonies for long periods of time.^24^ Disseminated tumour cells are static to a large extent, and their proliferation in metastatic sites is greatly inhibited because of their incompatibility with the surrounding microenvironment.^24^ To solve this problem, these cells activate a cellular nonautonomous mechanism. For example, the outgrowth of otherwise indolent disseminated tumour cells may depend on the activation and mobilization into the circulation of bone marrow‐derived cells and the subsequent recruitment of these cells to a metastatic site.^25^ Structural basis is a prerequisite for tumour metastasis, but so far, thorough research is still lacking.

## 4 MOLECULES IN THE PROCESS OF TUMOUR METASTASIS

Some kinds of endothelial adhesion molecules, such as integrin and selectin, can regulate the infiltration of T cells in the tumour microenvironment as long as desmosomes and tight junctions are present. However, little is known about the genetic mechanism of these molecules when they affect immune cells in cancer patients.

The expression of the Ras gene can be used as a G protein to participate in the signal transduction process of tumour cells.^26^ The ErbB gene can express epidermal growth factor receptor (EGF) and participate in signal transduction. In addition, there are other genes related to tumour metastasis, such as MTA1, and the FAKT c‐Src gene, which are also involved in the signal transduction process of tumour cells and affect their metastasis. The FAK gene, for example, encodes FAK protein, also known as adhesion kinase, a nonrecipient protein tyrosine kinase that regulates the adhesion, migration, survival, proliferation and differentiation of a variety of cells. The c‐Src gene encodes a receptor tyrosine kinase, which activates the receptor tyrosine kinase in squamous cell carcinoma when it binds to the growth factor. The activation of nuclear‐targeted FAK kinase leads to the depletion of T cells, the recruitment of regulatory T cells, such as CD8^+^ T cell, and the promotion of the immune escape of tumour cells. Furthermore, the activation of this kinase can affect the adhesion of plaques by affecting the activity of patchy enzymes.^27^ At the same time, activated c‐Src could induce the endocytosis of E‐cadherin, destroy adhesion, decrease adhesion ability and increase the capability of invasion. In gastric cancer cells, activated c‐Src can increase MMP‐2 and MMP‐9 expression and the degradation of the ECM, and play the role of VEGF‐C, thereby increasing the ability of gastric cancer cell metastasis through a series of processes.^28^

In addition to the related genes, cell adhesion molecules participate in the signal transduction of tumour cells. Many cell adhesion molecules have been discovered with a wide spectrum of responsibilities, including recruiting, activating, elongating and maintaining.^29^ More than 50 cell adhesion molecules have been found up to now, and they are divided into cadherins, integrins, selectins and the immunoglobulin superfamily CD44 according to their structural characteristics. Several tumour metastasis‐related molecules are briefly described below.

### 4.1 Cadherins

The classic cadherin is a multifunctional cell adhesion receptor. It is a multidomain, trans‐membrane protein in which the extracellular domain forms the homotypic, adhesive interaction while the intracellular domain interacts with the actin cytoskeleton through the catenin family of adaptor proteins.^30^ There are many subtypes of the cadherin family, including E‐cadherin, P‐cadherin and N‐cadherin. Taking E‐cadherin as an example, it has been shown that E‐cadherin is an inhibitor of tumour invasion and metastasis in various human tumour tissues.^31^ In a clinical study of oesophageal cancer cells, it has been confirmed that the expression of E‐cadherin affects the migration ability of cancer cells. When the expression level of E‐cadherin decreased, the migration ability of patients, mediated through PER‐2/per‐2 and HDAC‐1, was strong. The higher expression of PER‐2/per‐2 in metastatic oesophageal carcinoma cells inhibits the expression of E‐cadherin, while HDAC‐1 is involved in the inhibition of E‐cadherin induced by PER‐2 and blocks the ability of HDAC‐1 to inhibit the migration of oesophageal cancer cells.^32^

### 4.2 The integrin family

The integrin family is a heterodimer cell surface receptor family that perceives these changes and triggers a series of cellular responses by forming physical connections inside and outside the cell, thus allowing bidirectional ‘integration’ signals to control cell adhesion, migration, proliferation, survival and differentiation. At present, eighteen α subunits and eight β subunits have been reported, which can form at least twenty‐four heterodimer receptors.^33^

During local invasion, integrins can regulate multiple signals and affect the transmission of tumours in primary sites. When tumour cells escape into blood vessels or lymphatic vessels, they must adapt to survive without ECM adhesion. At this point, integrin controls the central role of growth and survival without relying on anchorage, as integrin β‐1 promotes prostatic anchorage‐independent growth,^34^ for example. Sowing a ‘metastatic niche’ requires specific recognition between cancer cells and their surrounding ECM. Since the integrin library expressed by a specific tumour cell may determine the ability to respond to a specific niche even in the absence of ligand binding to promote stemness, integrins may be key factors for tumour cells to home in the metastatic organ environment.^35^

### 4.3 Selectin

Selectin is a surface lectin that regulates the adhesion of leucocytes, platelets and endothelial cells in the bloodstream, including L‐selectin, E‐selectin and P‐selectin. These proteins are expressed in platelets and vascular endothelial cells. Among them, L‐selectin plays a good role in leucocyte recruitment, mediating inflammation and lymphocyte homing.^36^ E‐selectin and P‐selectin could regulate the metastasis of colon cancer cells, and the metastasis rate of SCID mice lacking these proteins decreased significantly.^37^

### 4.4 Growth factors

The formation of neovascularization can satisfy the substance supply of the tumour. Neovascularization is an essential condition for tumour growth and plays an important role in the process of tumour invasion and metastasis. Tumour cells host epithelial cells, endothelial cells and other cells and can secrete a variety of active factors to induce tumour angiogenesis. For example, growth factors secreted by tumour cells can stimulate this process, so these growth factors are called angiogenic factors, including heparin‐binding growth factors or fibroblast growth factor families, transforming growth factor α, vascular osmotic growth factor (VPF) and vascular endothelial growth factor (VEGF). These growth factors promote the proliferation of vascular endothelial cells and induce angiogenesis by combining with corresponding target cells.^38^ VEGF‐A interacts with VEGFR‐1 and VEGFR‐2 to mediate angiogenesis, while VEGF‐B and PIGF only have high affinity to VEGFR‐1. VEGF‐C and VEGF‐D combined with VEGFR‐2 and VEGFR‐3 regulate angiogenesis and participate in lymphangiogenesis.^39^ In a study of breast cancer, patients with higher VEGF expression have a poorer prognosis and earlier recurrence than tumour patients with lower VEGF expression after surgery.^40^

Epithelial to mesenchymal transition (EMT) is the highlight affecting changes in adhesion factors and related signalling pathways and promotes cell migration during tumour metastasis.^41^ In vitro model studies of tumour cell lines provide evidence that EMT occurs in the process of cancer invasion.^42^ The EMT can be triggered by different inducers, including a variety of growth factors, transforming growth factor‐β (TGF‐β), hepatocyte growth factor (HGF) and epidermal growth factor (EGF). For example, in the TGF‐β/SMAD signalling pathway, TGF‐β regulates cell function and has key roles in development and carcinogenesis. The intracellular effectors of TGF‐β signalling, the Smad proteins, are activated by receptors and translocate into the nucleus, where they regulate transcription.^43^ In addition, TGF‐β can also regulate the expression, secretion and activity of MMP‐2 and MMP‐9 and finally enhance the migration and invasion of the endothelial cells required for angiogenesis.^44^

### 4.5 MMPs

Proteolytic enzymes are required in several critical stages of tumour progression, such as the hydrolysis of ECM, which requires a series of specific proteolytic enzymes to complete the process. These proteolytic enzymes include MMPs, matrix‐degrading enzymes and cysteine proteases. For example, MMPs catalyse various proteins, including collagen, and matrix degradation, resulting in the remodelling of the structure of the ECM, making it more conducive to tumour cell migration and thereby promoting tumour metastasis. The major MMPs associated with tumour angiogenesis are MMP‐2, MMP‐9 and MMP‐14, followed by MMP‐1 and MMP‐7. MMP‐3, MMP‐7, MMP‐9 or MMP‐16 directly dissects the matrix‐bound vascular endothelial growth factor, leading to a change in the bioavailability of the modified VEGF molecule. MMPs can also cut the extracellular domain of cadherins in endothelial cells and destroy the intercellular junctions. Multiple angiogenesis promoters can induce the expression of MMPs in endothelial cells, and MMPs, in turn, enhance the biological activity of angiogenic factors. MMP substrates also include adhesion molecules and apoptosis‐mediated factors that regulate cell adhesion and change the ability of tumour cells to metastasize.^45^ In addition, the expression of karyotype MMP‐1 is detected in the stromal cells of breast cancer, and the total expression of MMP‐1 in breast cancer patients is associated with a poor survival rate.

## 5 RELATED CELLS IN THE PROCESS OF TUMOUR METASTASIS

**Figure 2:**
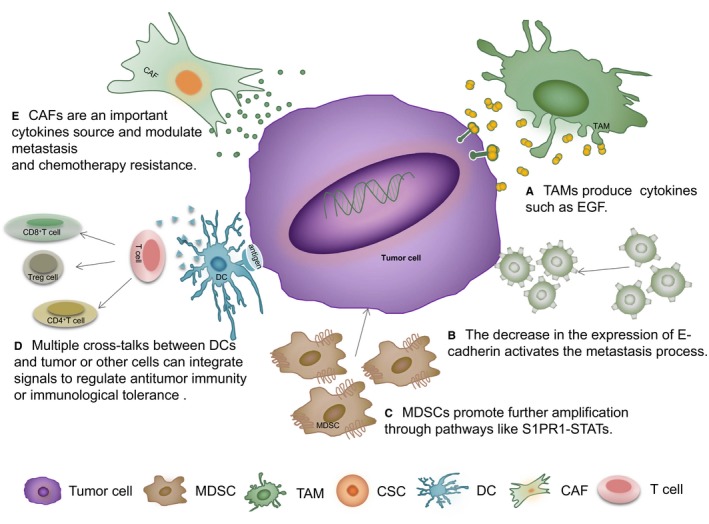
Interactions between cells in the tumour microenvironment. A, Tumour and CAFs‐derived cytokines can recruit macrophages and then produce a large number of associated cytokines, such as EGF, VEGF and TGF‐β, and the high expression of HIF‐1 and HIF‐2 promotes the formation of vascular mimicry. Angiopoietin Tie2 also makes some differences. B, The EMT of CTCs can promote the selective distal implantation of tumour. C, MDSCs in the pre‐metastatic microenvironment can promote further amplification through the S1PR1‐STATs signalling pathway. D, Tumour cells present autoantigens to DCs and regulate T cell differentiation, thus affecting tumour development through Treg cells, CD4^+^ T cells and CD8^+^ T cells by immune actions. E, CAFs are abundant in tumour microenvironment and an important cytokines source

TAMs and CTCs play a catalytic role in both the local invasion of tumour cells into the vascular cavity and the survival of tumour cells under blood flow conditions. Tumour‐derived CSF‐1 can recruit macrophages to home and produce a large number of associated cytokines, such as EGF, VEGF, TGF‐β, to enhance cell invasiveness 46 (Figure 2A). CSF‐1 and TAM‐derived EGF promote the production of each other and form a positive circulation that magnifies the promotion effect.^47^ In addition, the anoxic microenvironment of tumours can up‐regulate the expression of hypoxia‐inducible transcription factors (HIF‐1 and HIF‐2) in TAMs, which subsequently helps to form tumour blood vessels, lymphatic vessels and vascular mimicry.^48^, ^49^ The expression of Tie2 increased during the chemotaxis of tumour tissues and the production of TAMs.^49^ TAMs also help to form the tumour immunosuppressive environment. IL‐10, TGF‐β and PGE‐2 contained in the tumour microenvironment can inhibit the expression of MHC class II molecules on the surface of TAMs, thus hindering the antitumour effect guided by T cells. And TAMs themselves also exert their inhibitory function through many approaches, keeping the tumour microenvironment in the safe state of immune escape.

CTCs are cells similar to primary tumour cells and are usually used as markers of distant metastasis. The most important characteristic of CTCs is that they undergo EMT reversible transformation (Figure 2B) and the same is true for CSCs. EMT is an activity based on the CTC plasticity that tumour cells divert between the two phenotypes: an epithelial type and a mesenchymal type, which can promote the selective distal implantation of tumour.^50^ The decrease in the expression of E‐cadherin induces the falling off of tumour cells. It also activates the metastasis process together with the infiltration of cells into the blood vessels.^51^ In turn, when CTC is reversed in distal organs, the adhesion between cells increases, benefiting the plantation of tumour cells in distal organs and the establishment of metastatic foci.^52^ According to the self‐implantation theory, CTCs can also release signals before tumour invasion to stimulate the tumour cells to return to the primary site for self‐implantation depending on the SCUB3 pathway.^53^

CSCs are defined as cancer cells with the stem‐cell‐like ability. The original CSCs may participate in metastasis or a new type of CSC derived from the first one or another cell in the tumour that acquires metastatic traits could do so. The formation of such a ‘metastatic CSC’ might be observed as EMT.^54^ Once CSCs have metastasized, they must contribute to neoplastic growth in the new location where a smaller percentage of CSCs is contained than the primary tumour.^55^ According to the mathematical model, while the intraspecific interactions between CSCs and the rest of a tissue are inhibitory, the interspecific interactions stimulate tumour growth, suggesting that stem cells needing differentiated cells to reinforce their niches, and phenotypic plasticity favouring the de‐differentiation of differentiated cells into CSCs.^56^ As a result, CSCs are critical to the treatment of a variety of solid cancers. For example, recently studies show that CD271, a cancer stem cell biomarker of HPC, can be a promising therapeutic target.^57^ However, CSCs can be involved in cancer recurrence owing to their tumorigenic properties and supposed resistance to many conventional therapies.^54^ Breast CSCs grown in culture are resistant to chemotherapeutic agents 58 and are much harder to be eliminated than other cancer cells.^59^

When tumour cells prepare to intravasate into the parenchyma of distant tissue, myeloid‐derived suppressor cells (MDSCs) may facilitate this process. These cells mainly help to form a prosurvival environment for metastasis and prepare for the subsequent colonization and reproliferation of a large number of tumour cells. Under the action of many tumour‐derived factors, medullary tumour cells proliferate and mobilize into the blood to act on distal target organs. MDSCs in the pre‐metastatic microenvironment can promote further amplification through the S1PR1‐STATS signalling pathway and help tumour cells penetrate into the circulatory system (Figure 2C). S1PR1 is a G protein‐coupled receptor of lysophosphatidyl 1‐sphingosine, and its expression is increased in STAT3^+^ tumour cells. The upregulation of the S1pr1 gene can activate STAT3 and promote tumour growth and metastasis. Some studies have shown that S1PR1 leads to the sustained activation of STAT3 and that STAT3 can induce the expression of the S1pr1 gene. Both activities promote the expression of STAT3 in tumour cells and play an important role in tumour progression.^60^, ^61^, ^62^ Subsequently, MDSCs can also mediate changes in inflammatory and immunosuppressive components in the microenvironment before metastasis. Inflammation has its own place in the cancer process and provides the necessary conditions for tumour development, which means that MDSCs indirectly affect the development of tumours by secreting related factors and proteins to construct the pre‐metastatic microenvironment of inflammation and immunosuppression. Different subsets of MDSCs have different mechanisms of forming the condition of immunosuppression. Some studies have also shown that MDSCs have the capability to cause vascular hyperosmosis 63 and promote the migration and homing of tumour cells.^64^

Dendritic cells (DCs) are heterogeneous and specialized antigen‐presenting cells (APCs) that have an unequalled capacity to initiate primary immune responses. Growing evidence suggests multiple cross‐talks between DCs, and other cells from tumour microenvironment can contribute to antitumour immunity or immunological tolerance by regulating T cell differentiation and responses 65 (Figure 2D). Tumour cells present autoantigens to DCs and activate T cell differentiation, thus affecting tumour development through Treg cells, CD4^+^ T cells and CD8^+^ T cells by immune actions.^66^ Nitric oxide synthase (NOS2)‐ and tumour necrosis factor (TNF)‐producing DCs are important for adoptively transferred CD8(+) cytotoxic T cells to destroy tumours.^67^ In addition, immunosuppressive cells such as Treg cells can secrete immunosuppressive molecules that are known to induce DC dysfunction.^68^

Cancer‐associated fibroblasts (CAFs) are important components of the tumour microenvironment and appear in almost every stage of invasion. The toxic tumour microenvironment may induce the phenotypic differentiation of multiple resident precursors into CAFs.^69^ CAFs can function as a regulator of immune cell recruitment, and the recruitment of monocytes by CAFs was mediated by MCP‐1 as well as SDF‐1 cytokines.^70^ CD10^+^ GPR77^+^ CAFs promote tumour formation and chemoresistance by providing a survival niche for CSCs.^71^ Recent studies revealed that CAFs can contribute to tumour proliferation, invasion and metastasis via promoting secretion of various factors, tumour‐stroma interaction, angiogenesis and chemoresistance 72 (Figure 2E).

In general, all the various cells mentioned above enhance the ability of tumour cells to metastasize and act at different stages of invasion by directly secreting proteins or indirectly make contributions with the help of other cells. In addition, the microfilament microtubules and mesenchyme in the cytoskeleton are also associated with the tumour, but to what extent can these factors influence the cancer progression and the detailed mechanisms are still unclear, which calls for further study.

## 6 THE PRESENT SITUATION AND THE PROMISE OF TARGETING METASTASIS IN EMERGING TUMOUR THERAPY

The current treatments for tumour limited mainly in surgery, chemotherapies and radiotherapies. The interdisciplinary collaboration among nanotechnology, biology, chemistry, pharmacology, oncology and other disciplines has led great changes in these three primary therapy.^73^

Targeted drugs are imperative in the treatment of cancer. As molecules or their preparations endowed with targeting ability, they form a relatively high concentration of drugs in the target area, so as to improve the efficacy and inhibit the toxic and side effects to normal tissues and cells.^74^ Bevacizumab, for example, an antibody neutralizing monoclonal against VEGF or its receptor, in combination with other chemotherapeutic agents has been approved to be the first line therapy in metastatic colorectal carcinoma (mCRC) by the FDA. Small molecule tyrosine kinase inhibitors of VEGF receptors, soluble VEGF receptors which act as decoy receptors for VEGF, and ribozymes which specifically target VEGF mRNA can also block VEGF pathway.^75^ Olimab, a human IgG1 antibody that binds to platelet‐derived growth factor receptor α (PDGFR‐α), is a new drug for soft tissue sarcomas. PDGFR‐α receptors are also detected in some tumours and stroma cells, including sarcomas.^76^ In recent years, new targets are adopted at a rapid and exponential rate, following by new tumour‐targeting drugs. Studies show that a histone methyltransferase nucleoreceptor‐binding SET domain protein 2 (NSD2), which plays a key role in the apparent genetic control of gene expression, is suggested to be targeted for the treatment of advanced prostate cancer.^77^ Recently, through the analysis of multiple clear cell renal cell carcinoma (ccRCC) cases, the evolutionary potential of MMP is proposed as a biomarker for intervention and detection.^78^ The most exciting development in recent years is the breakthrough of chimeric antigen receptor (CAR)‐T cell therapy. As one of the adoptive immunotherapies, CAR‐T cell therapy aims at returning the edited white blood cells back to the patient.^79^ Two CAR‐T therapeutic drugs, Kymriah from Novartis and Yescarta from Kite Pharma, have been approved to treat leukaemia and lymphoma, respectively, in the historic year of CAR‐T, 2017.

Aside from things above, latest methods for cancer treatment are emerging in an endless steam. For example, Chitosan‐modified PLGA polymeric nanoparticle system kills cancer cells and reduces pain by facilitated to apoptosis, especially for metastatic cancers.^80^ Photothermal therapy (PTT) is another emerging tumour therapy by thermal ablation of tumour cells.^81^ In PTT process, a photosensitizer converts the light energy to heat energy, using the temperature evolution to kill tumour cells and avoid significant side effects on normal cells because tumour cells have a lower heat tolerance than normal ones.^82^ Additionally, genetically engineered T cell with the addition of genetic material, including cytokines and cytokine receptors, enhances receptor affinity and functional avidity of the genetically engineered T cells and has got promising results in current clinical trials.^83^ Oncolytic virus also works, and oncolytic vectors are designed for improved tumour specificity, intratumoural spread, therapeutic gene delivery and especially as targeted cancer immunotherapeutics.^84^

The field of cancer immunotherapy has recently received a significant attention. The treatment of targeted tumour metastasis prevents or slows down tumour metastasis, which prolongs the life of tumour patients and improves the clinical survival rate. However, it is still difficult for some of the targeted drugs to focus on tumour tissues and then affect other healthy tissues, resulting in adverse reactions due to the lack of specificity. More research is needed on the safety, specificity and stability of targeted therapy for tumour metastasis. In addition, the combined targeted delivery of treatments is an important method to enhance the therapeutic efficiency and reduce adverse side effects for current therapies.^85^ More attention should be paid to the design of optimal combination tumour microenvironment targeting therapy in the future.

## 7 CONCLUSION AND PERSPECTIVES

In this paper, we discussed the mechanisms of promoting tumour metastasis from the genetic, molecular, cellular and structural levels according to the time sequence of tumour metastasis and invasion. It is clear that the mechanisms are not independent, and the specific substances of each time course have many similarities. The molecules or cells that interact with each other among different pathways that are cross points, forming a network to coordinate tumour metastasis. Tumour metastasis is a major cause of tumour death. Controlling tumour metastasis can prolong the survival time and improve the quality of life of cancer patients. Therefore, studies of tumour metastasis can provide a variety of ideas for cancer treatment. Since there have already been a number of targeted tumour therapy drugs or treatment methods, anticancer drugs with more targeted, higher safety trends are developing. However, all studies have shown that homologous molecules may have expression in different tissues, that is, their expression in tumour organs is not special enough to be safe targets, and the origin point is still not appearing. Drugs manage to not only kill cancer cells but also destroy normal tissues and reduce the normal functioning of the body simultaneously. Furthermore, the same molecule may present the opposite functional characteristics at different stages of tumour development and this tendency usually hard to estimate, pulling one hair and affecting the whole body. When we are looking at an old tree, we are still doing research on its broomy branches and looking forward to digging into its main root. Generally, the research of tumour metastasis is developing continuously, but it is still not enough to support antitumour therapy. We hope that the study of tumour metastasis will not only broaden the number of molecules but also deepen the research on each related substance and determine the ultimate key point of tumour metastasis.

## CONFLICT OF INTEREST

The authors declare that they have no conflicts of interest to this work.

## AUTHORS' CONTRIBUTIONS

All authors wrote the manuscript and ZF designed the figures; JL and XZ provided guidance and revised this manuscript. All authors approved the final manuscript.
